# Remote tissue immune priming in allergic disease

**DOI:** 10.1038/s41385-020-0328-0

**Published:** 2020-07-27

**Authors:** Niki D. Ubags

**Affiliations:** Faculty of Biology and Medicine, Service de Pneumologie, CHUV, University of Lausanne, Lausanne, Switzerland

The mechanisms underlying co-occurrence of allergic diseases, such as atopic dermatitis, food allergy, and atopic asthma, are poorly understood. In this commentary on the manuscript entitled “Remote allergen exposure elicits eosinophil infiltration into allergen nonexposed mucosal organs and primes for allergic inflammation”, the author highlights the implications of intertissue communication for the development and progression of the “atopic march”.

The development of a series of atopic disease indications in early childhood is often referred to as the “atopic march”, a framework that proposes a progression from atopic dermatitis and food allergies in infants to allergic rhinitis and allergic asthma in children.^1^ Allergen sensitization is a first step toward the development of allergic diseases. An impaired skin barrier function can lead to increased allergen penetration through the skin and consequently promote Th2 responses locally (in the skin), but also systemically and at distal mucosal sites including the gut and the lung. Moreover, oral sensitization and the development of food allergy can also enhance the risk of allergic asthma and allergic rhinitis. The mechanisms underlying co-occurrence of allergic diseases are poorly understood. Advances in understanding the immunological processes underlying the sequence of events in the atopic march will aid in development of early interventions, which may halt the progression of allergic diseases.

The description of the gut–lung axis and the gut–skin axis has contributed to development of the notion that interorgan crosstalk could be involved in the co-occurrence of allergic diseases. However, until recently, the potential for remote tissue immune priming in the absence of an allergen challenge in the remote tissue had not been explored. Olbrich et al.^2^ set out to answer this question and determined whether local allergen exposure influences eosinophil frequency and phenotype in remote mucosal tissues using a murine model of systemic allergen sensitization (alum-ovalbumin) followed by either skin, gut, or lung challenge with ovalbumin. Allergen challenge on the skin, in the gut, or in the lungs did not only induce a local allergic inflammatory response characterized by local eosinophil influx, but was found to also initiate increased eosinophil frequencies in distant nonallergen exposed mucosal tissues. Even though circulating eosinophil levels were similar following the different exposures, exposure of the airways to allergen induced a stronger eosinophil infiltration in the gut compared with the intestinal eosinophil infiltration induced by allergen exposure on the skin. Moreover, intestinal allergen exposure led to a more robust eosinophil accumulation in the lungs, compared with the lung response observed following skin allergen challenge. This suggests that the lung–gut axis elicits a more robust response when compared with the skin–gut axis in promoting eosinophil infiltration into remote nonallergen exposed tissues.

It remains to be determined whether the cytokine and chemokine milieu of the gut and lung is distinct following allergen challenge on the skin, as these cues are imperative for eosinophil recruitment, phenotype, and function.^3^ Moreover, epithelial barrier function of subjects with allergic skin and lung disease is suggested to be abnormal,^4^ but the extent to which epithelial barrier function in remote nonallergen exposed mucosal tissues can be impacted by systemic eosinophilia, and whether these responses differ between mucosal tissues, is currently unknown. Recent evidence indicates that age and microbiome determined coordination of tissue immune cells controls the nature and severity of skin and lung allergic disease, and that the rules governing disease manifestation are determined by the immunological status of the barrier tissue.^5^ Specifically, formation of the skin microbiome during development drives cutaneous chemokine production and seeding of the tissue with antigen presenting cells (APCs) and thus immune maturation, which consequently allows mature APCs to localize inflammation to the site of exposure. The rules governing the lung response can be different from those driving skin and gut responses. This is in line with a recent observation where intestinal helminth infection was shown to influence lung responses (in the absence of a lung challenge) through IL-17-mediated dampening of IFNγ, which consequently enhanced Th2 responses.^6^

## Consequences of remote tissue eosinophil seeding following allergen challenge

Alterations in tissue immune cell composition during homeostasis and immune priming can enhance predisposition to disease. Allergen exposure can induce systemic Th2 priming and enhance eosinophilopoiesis. Moreover, enhanced tissue eosinophil levels are often the result of activated eosinophils from the bloodstream. Under homeostatic conditions, intestinal eosinophils are phenotypically different from blood eosinophils and localization within the tissue can be determined based on the expression of surface markers including CD11b and CD11c.^7^ Olbrich et al. demonstrated that remote allergen-elicited intestinal eosinophils acquired tissue-specific markers appropriate to their respective localization within the tissue, suggesting that they exhibit a resident intestinal tissue eosinophil phenotype.^2^ In contrast, intragastric allergen exposure led to the occurrence of two different eosinophil phenotypes in the lung, the first, resident homeostatic eosinophils, and the second phenotype, activated inflammatory eosinophils. The latter were characterized by increased levels of CD11c and SiglecF. The shift from a resident to an inflammatory phenotype has been reported for recruited eosinophils in the setting of allergic airway inflammation and was characterized by enhanced chemokine receptor (*Ccrl2, Ccr2*, and *Ccr5*) and integrin (*Itgae, Itgb1*, and *Itgb5*) expression.^8^

To further dissect the mechanisms underlying allergic disease co-occurrence, it is imperative to enrich our understanding of the involvement of enhanced tissue eosinophil numbers and altered eosinophil phenotypes in remote tissues. Interestingly, the inflammatory eosinophils in the lung following intragastric allergen challenge was accompanied with airway mucus production,^2^ suggesting there is an allergic inflammatory response ongoing in the lung that exhibits features of allergic asthma. To determine whether remote allergen exposure could exacerbate an allergic airway inflammatory response, Olbrich et al. exposed mice that were orally or cutaneously challenged with ovalbumin to an additional house dust mite allergen challenge in the lungs. They observed a more robust allergic response in the lungs from mice previously challenged in the gut or the lung,^2^ suggesting that remote tissue eosinophil seeding can exacerbate allergic disease.

The duration of the remote allergen-induced increase in tissue eosinophils was dependent on the mucosal tissue affected, with intestinal eosinophil numbers still being significantly different after 11 days, whereas lung eosinophil levels returned to baseline.^2^ Tissue resident eosinophils exhibit increased survival upon migration into the tissue. Moreover, the half-life of intestinal eosinophils is longer compared with lung eosinophils,^9^ which may be driven by the tissue-specific microenvironment. The question that remains is whether enhanced tissue eosinophils are required for the development of an exacerbated allergic airway response and whether there is a long-term consequence for predisposition to exacerbated allergic inflammation even when tissue eosinophil levels return to baseline.

## Concluding remarks and future directions

The importance of interorgan crosstalk has been highlighted by studies reporting the existence of the gut–lung axis and the gut–skin axis. However, immune homeostasis and priming in remote mucosal tissues that are not directly targeted in one specific allergic disease model have not received much attention. Although there is evidence for altered immune homeostasis and immune priming leading to exacerbated allergic responses in remote tissues,^2^ the underlying mechanisms remain to be elucidated. In doing so, it is important to focus on the origin of the infiltrating eosinophils, regulation of eosinophil recruitment and apoptosis, alterations in the local and systemic cytokine and chemokine milieu, and the long-term effects of alterations in immune homeostasis and priming on allergic disease development and progression. This knowledge will aid in the identification of new targets for intervention strategies aimed at halting co-occurrence of allergic diseases as observed in the “atopic march.”
